# Supplementary material for: Effects of a Combination of Polynucleotide and Hyaluronic Acid for Treating Osteoarthritis
Source: Int J Mol Sci. 2024 Jan 30;25(3):1714. doi: 10.3390/ijms25031714 (PMC10855695; doi:10.3390/ijms25031714)
Supplement: Supplementary file 1 [file ijms-25-01714-s001.zip › Supplementary figure S1.pdf]

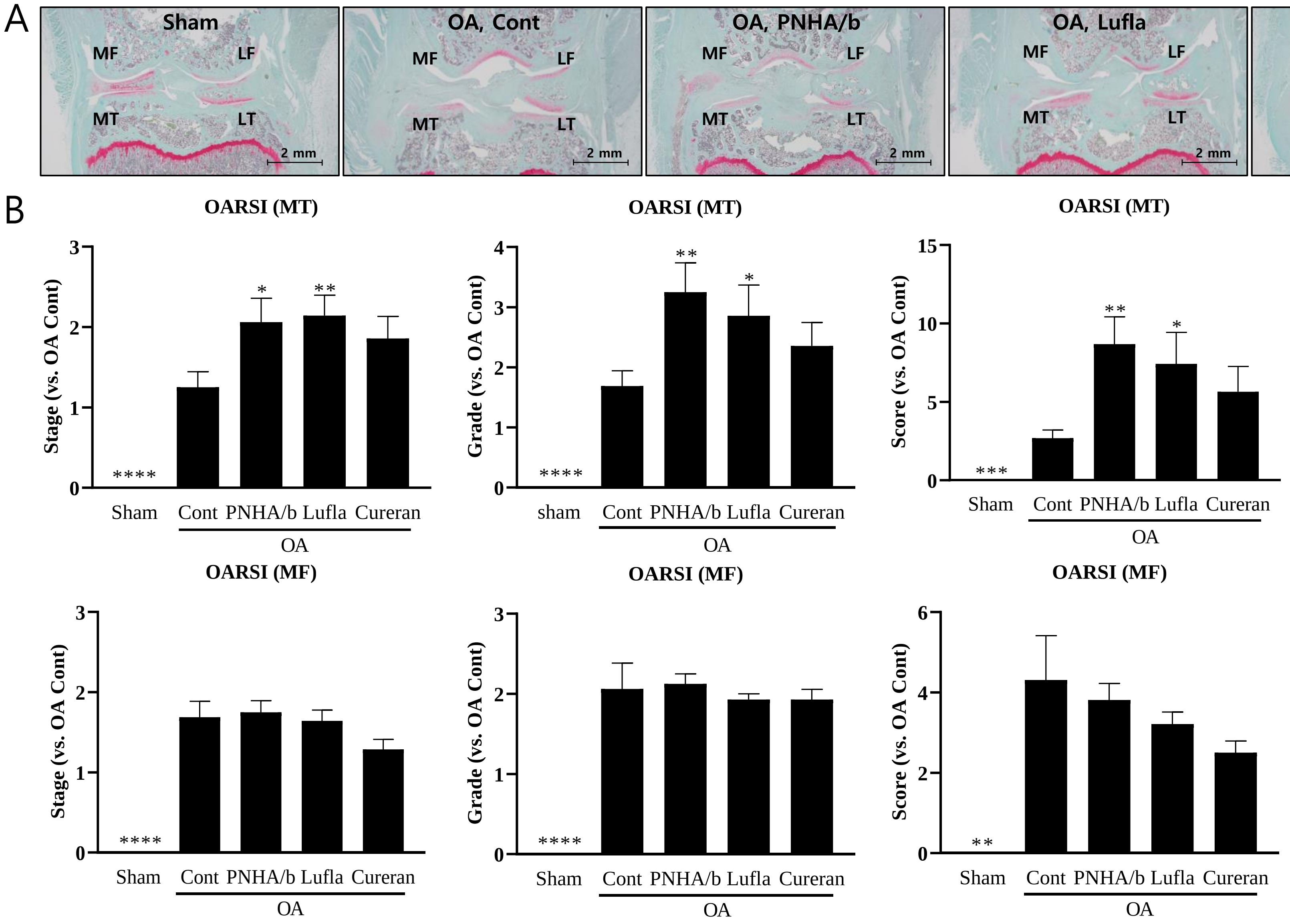

Supplementary Figure S1.

(A) Safranin O-Fast Green Staining. (B) OARSI score and grade in tibia and femur. n = 10 – 12 per group. Sham, sham operation; OA, osteoarthritis; Cont, control vehicle; PNHA/b, polynucleotide (2%) with hyaluronic acid (0.5%); HA, hyaluronic acid (0.5%). Relative to OA control: *$p < 0.05$, **$p < 0.01$, ***$p < 0.0001$ (unpaired t-test). The data are expressed as the mean ± SEM.
