# Supplementary material for: Effects of a Combination of Polynucleotide and Hyaluronic Acid for Treating Osteoarthritis
Source: Int J Mol Sci. 2024 Jan 30;25(3):1714. doi: 10.3390/ijms25031714 (PMC10855695; doi:10.3390/ijms25031714)
Supplement: Supplementary file 1 [file ijms-25-01714-s001.zip › Supplementary figure S2.pdf]

A

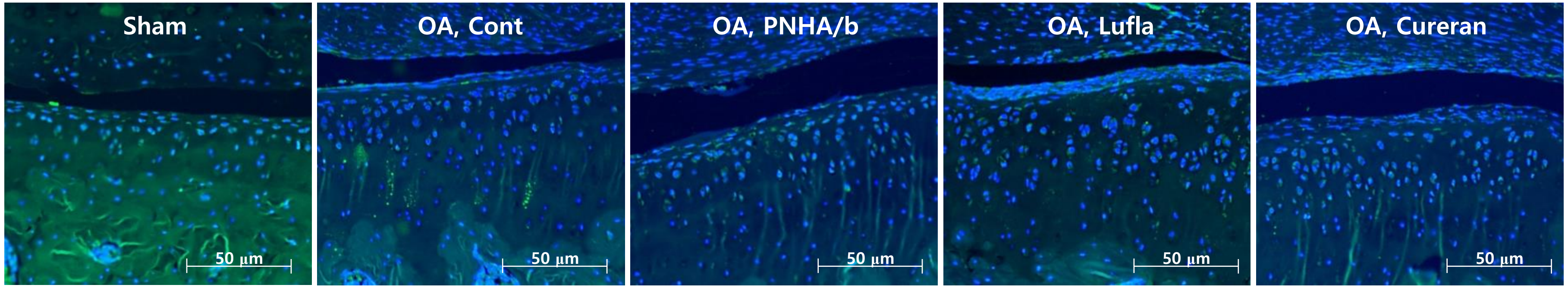

B

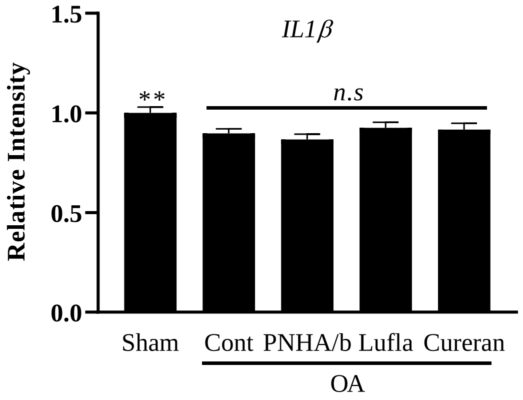

Supplementary Figure S2.

(A) IL1β immunofluorescence staining in medial joint cartilage. (B) Quantification of IL1β staining amount in articular cartilage. n = 8 – 13 per group. Sham, sham operation; OA, osteoarthritis; Cont, control vehicle; PNHA/b, polynucleotide (2%) with hyaluronic acid (1%); Lufla, Hyaluronic acid (1%); Cureran, polynucleotide (2%). Relative to OA control: $**p < 0.01$, (unpaired t-test). n.s, not significant. The data are expressed as the mean ± SEM.
